# Correlation of the Upper Nasolabial Angle With Skeletal Patterns and Nasal Morphology in Indian Adults: An Observational Study

**DOI:** 10.7759/cureus.68857

**Published:** 2024-09-07

**Authors:** Seema Gupta, Adeel A Bajjad, Anil Sharma, Sheikh Ummae Hani, Anurag Kumar, Vatsal Pandey

**Affiliations:** 1 Department of Orthodontics, Kothiwal Dental College & Research Centre, Moradabad, IND

**Keywords:** correlation, morphology, nasal, nasolabial, skeletal

## Abstract

Introduction: The upper nasolabial angle (UNLA) is an important determinant in clinical diagnosis and treatment planning. Therefore, the primary objective of this study was to assess the correlation between various skeletal parameters and nasal morphology in an Indian cohort, particularly UNLA. The secondary objectives were to evaluate sex differences in nasal morphology, optimal cut-off values of the UNLA for skeletal malocclusions, and differences in nasal morphology in different skeletal malocclusions.

Materials and methods: This retrospective study used 162 pre-treatment lateral cephalometric records of patients with skeletal Class I, Class II, and Class III relationships. Group 1 consisted of 68 skeletal Class I patients (ANB angle of 2-4^0^), Group 2 consisted of 68 skeletal Class II patients (ANB angle > 4^0^), and Group 3 consisted of 26 skeletal Class III patients (ANB angle of less than 2^0^). Each group was further subdivided according to the growth pattern into low (horizontal), average, and high (vertical) growers. The angle between the sella-nasion plane and mandibular plane (SN-GoGn angle) was used to further divide the groups according to their growth pattern into a horizontal growth pattern (SN-GoGn angle < 28^0^), an average growth pattern (SN-GoGn angle of 28-36^0^), and a vertical growth pattern (SN-GoGn angle > 36^0^). The study sample was also divided into males and females to assess gender differences. Spearman’s correlation coefficient, multinomial logistic regression analysis, Mann-Whitney U test, and Kruskal-Wallis tests were used. Receiver operating characteristic curves were used to obtain the optimal cut-off values for UNLA.

Results: The UNLA showed a positive correlation with the sagittal position of the maxilla and a negative correlation with the sagittal position of the mandible and palatal plane (PP) inclination angle. Statistically significant sex differences were observed in nasal length, nasal depth, and lower anterior facial height. The optimal cut-off values for the UNLA angle in the class II skeletal pattern were ≥ 20^0^, 16-20^0^ for skeletal class I, and ≤ 16^0^ for skeletal class III patients. None of the skeletal and nasal parameters were reliable predictors of the skeletal pattern type.

Conclusion: The present study showed that the UNLA increased in the skeletal class II pattern with the downward inclination of the PP. Patients with skeletal class III malocclusion had an upward-canted nasal tip.

## Introduction

Facial balance in orthodontics is established by morphological relationships and ratios of the nose, lips, and chin. The position of the nose at the center of the face contributes significantly to facial aesthetics. This is evident in the notable improvement in facial aesthetics after minor rhinoplasty. A straight nasal dorsum with the dorsal cartilage and nasal tip cartilage positioned above the nasal tip, creating a supra tip break, is essential for achieving the ideal nasal proportion. An ideal nose should complement other facial features, with variations in nasal characteristics observed across different races and other facial attributes [1]. Orthodontic procedures, such as retraction of protruded incisors and orthognathic maxillary and mandibular surgeries, affect nasal morphology, particularly the nose prominence and nasolabial angle [2].

The nasolabial angle has two components: the lower nasolabial angle (LNLA), which reflects the upper lip angulation, and the upper nasolabial angle (UNLA), which reflects the nasal angulation. Nehra and Sharma observed that upward nasal tip inclination was significantly negatively correlated with palatal plane (PP) inclination [3]. However, they did not evaluate the influence of different skeletal patterns on the UNLA. Orthodontic retraction of the upper incisors in patients with upward nasal tip angulation can further deteriorate their facial aesthetics; therefore, it is important to know the factors that influence UNLA. According to a study by Bhardwaj et al., the nasal length increased in skeletal class III, and sexual dimorphism was also observed in nasal morphology [4]. The importance of the LNLA in orthodontics has been studied extensively in the literature; however, very few studies have been conducted on the UNLA.

According to a systematic review by Jankowska et al., nasal parameters are correlated with skeletal class, nasolabial angle, position of the upper incisors, and maxillary inclination [2]. Most of these studies were conducted in Western populations, where it was noticed that nasal length, prominence, and form are associated with the height and length of the maxilla and mandible in Anatolian Turkish adults [5]. Linear nasal parameters varied between the male and female groups in all skeletal malocclusions in the Turkish population [6], and significant ethnic differences were noted in nasal morphology [7]. Considering the ethnic and racial differences in the Indian population compared to Western cohorts, it is imperative to assess the role of different factors affecting nose morphology, particularly the UNLA, in different skeletal malocclusions in Indian-based populations. To the best of our knowledge, no study has assessed the correlation between various skeletal parameters and nasal morphology in an Indian population. Therefore, the primary objectives of this study were to assess the correlation between various skeletal parameters such as sagittal and vertical position, length of maxilla and mandible, PP inclination, and nasal morphology, particularly the UNLA, and predictors of the type of skeletal pattern in Indian cohorts. The secondary objectives were to assess the gender differences in nasal morphology, optimal cut-off values of UNLA for skeletal malocclusions, and differences in nasal morphology in different skeletal malocclusions.

## Materials and methods

Study design and setting

This retrospective, single-center, cross-sectional observational study was approved by the institutional ethics committee (KDCRC/IERB/02/2022/39) and was conducted according to the principles of the Declaration of Helsinki. The Strengthening the Reporting of Observational Studies in Epidemiology (STROBE) guidelines were followed for this study. Written informed consent to use the records of the patients for research purposes and maintaining confidentiality is routinely taken as a routine protocol of the department. The study sample consisted of pre-treatment lateral cephalogram records of 162 patients at the Department of Orthodontics between August 2015 and May 2022.

Participants and sample size calculation

The sample size was calculated using the G Power software version 3.2.9.7. The power analysis showed that a total sample size of at least 162 subjects would give a 95% probability of detecting a real difference between the intervention groups at a statistically significant level of 5% by keeping the minimal effect size of 0.3 in nasal depth parameter [8].

**Figure 1:**
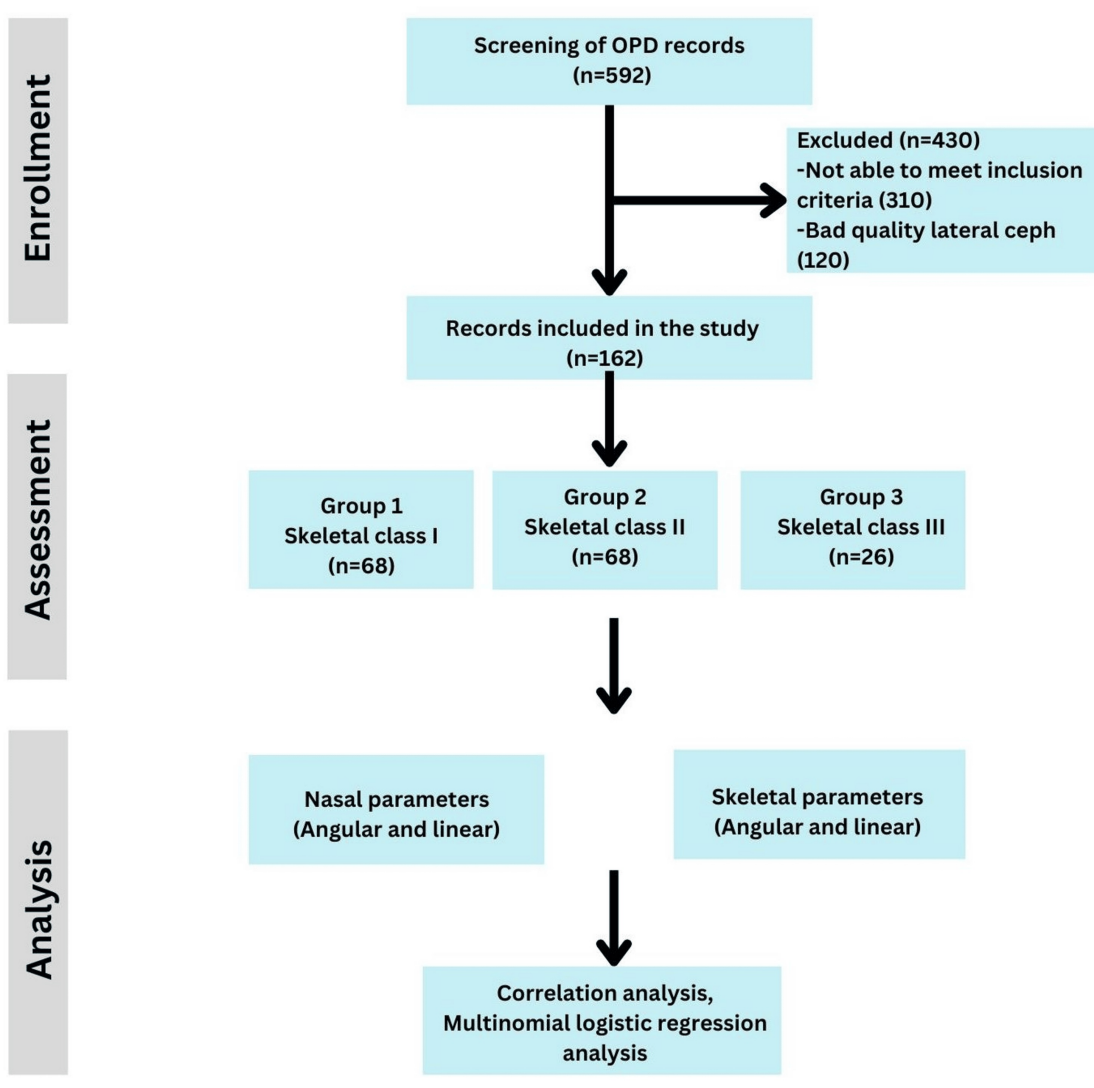
Study flow diagram. Ceph: Cephalogram; OPD: Outpatient Department

The study sample was selected based on the following eligibility criteria: age range-18-30 years, Indian origin, presence of good quality pre-treatment lateral cephalograms, no history of orthodontic treatment, absence of any congenital deformity, facial injury or trauma, nasal surgeries or trauma, enlarged adenoids, sinusitis, and severely decayed or missing upper anterior teeth were included in the study. Five hundred and ninety-two records were initially screened, and 162 were selected for the study based on the eligibility criteria. The study design is illustrated in Figure 1.

The study records were divided into three main groups according to the maxillomandibular relationship and subgroups based on their growth patterns. Group 1 consisted of 68 skeletal Class I patients (ANB angle of 2-4^0^), Group 2 consisted of 68 skeletal Class II patients (ANB angle > 4^0^), and Group 3 consisted of 26 skeletal Class III patients (ANB angle of less than 2^0^). Each group was further subdivided according to growth pattern into low (horizontal), average, and high (vertical) growers. The angle between the sella-nasion plane and mandibular plane (SN-GoGn angle) was used to further divide the groups according to their growth pattern into a horizontal growth pattern (SN-GoGn angle < 28^0^), an average growth pattern (SN-GoGn angle of 28-36^0^), and a vertical growth pattern (SN-GoGn angle > 36^0^). The study sample was also divided into males and females to assess gender differences.

Methodology

Lateral cephalograms were acquired in the natural head position using a KODAC 8000 C Digital Panoramic and Cephalometric system set at 70 KV and 10 MPa. Consistent exposure parameters were used to capture all the cephalograms. Good-quality lateral cephalograms were obtained and traced with a 0.3-mm lead pencil on acetate tracing paper. In the current study, lateral cephalometric radiographs were used to assess nasal characteristics because of their routine application in diagnostic and therapeutic decision-making processes. 

**Figure 2:**
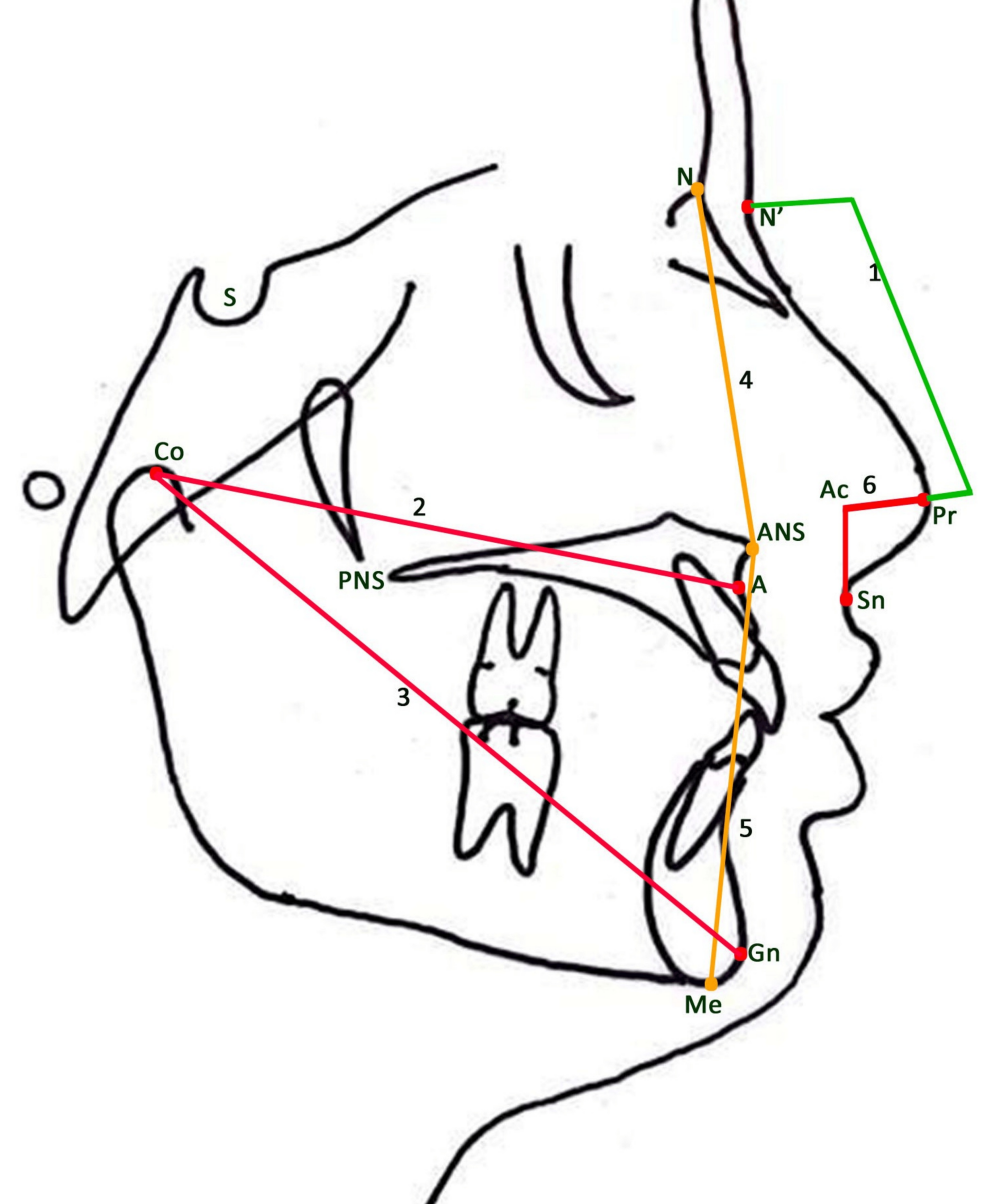
Linear measurements. 1. Nasal length (N’-Pr); 2. Effective maxillary length (Co-A); 3. Effective mandibular length (Co-Gn); 4. Upper anterior facial height (N-ANS); 5. Lower anterior facial height (ANS-Gn); 6. Nasal depth (Ac-Pr). This figure is the author's original work.

**Figure 3:**
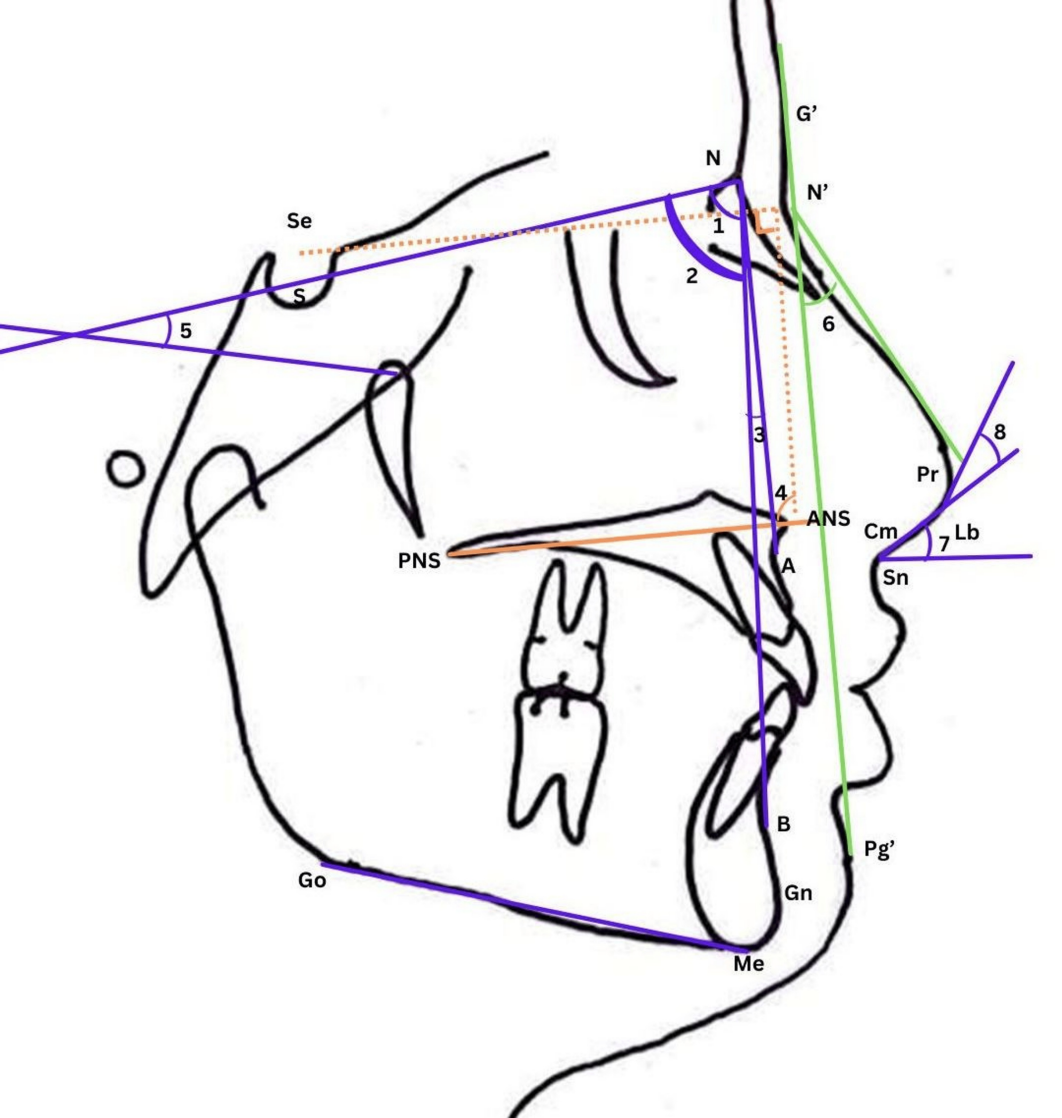
Angular measurements. 1. SNA; 2. SNB; 3. ANB; 4. Palatal plane inclination; 5. SN-GoGn; 6. Nasofacial angle; 7. Upper nasolabial angle; 8. Nasal tip angle (Cm-Lb). This figure is the author's original work.

The skeletal parameters for maxilla were SNA angle (maxillary sagittal position), Co-A in mm (effective maxillary length), N-ANS in mm (upper anterior facial height), and PP inclination angle (inclination of PP with respect to the cranium). The skeletal parameters of the mandible were the SNB angle (mandibular sagittal position), Co-Gn (effective mandibular length), SN-GoGn angle (mandibular inclination with respect to the cranium), and lower anterior facial height (ANS-Gn). Maxillo-mandibular parameters (ANB angle) were also recorded. The nasal parameters included the nasal length, depth, UNLA, Cm-Lb (columellar-lobular), and nasofacial (NFL) angle. All landmarks and six linear and eight angular measurements are described in Table 1 (Figures 2, 3).

**Table 1:** Definition of landmarks and measurements used in the study.

Sr.no	Landmarks	Description
1	N	Nasion (anterior most point on frontonasal suture)
2	S	Sella (midpoint of sella tursica)
2	A	The most concave point between anterior nasal spine (ANS) and alveolar bone overlying the maxillary incisor root
3	B	The most concave point between the most prominent point on the chin (Pog) and alveolar bone overlying the mandibular incisor root
4	Go	Gonion (the constructed point at the intersection of the ramal plane with the mandibular plane)
5	Gn	Gnathion (Most antero-inferior point on chin)
6	Co	Condylion (Most postero-superior point on the condyle)
7	PNS	Posterior nasal spine
8	ANS	Anterior nasal spine
9	Me	Menton (the lowermost point on chin)
10	N’	Soft tissue nasion (the point of greatest concavity in the midline between the forehead and the nose)
11	Pg’	Soft tissue pogonion (the most prominent point on soft tissue chin)
12	Se	The midpoint at entrance of sella turcica
13	Pr	Pronasale (point at nasal tip)
14	Cm	Columella point (the most anterior point of the columella)
15	Sn	Subnasale (the point at which the nasal septum forms an angle with the philtrum)
16	Lb	Infratip lobular point (the area of the nose bounded by the tip-defining points superiorly, and the columella caudally)
17	G’	Glabella point (the most prominent point on forehead)
18	Ac	It is a constructed point by extending Sn point upward
19	PP	Palatal plane (line joining ANS and PNS)
20	SN-GoGn	Angle between SN plane and mandibular plane which is the plane joining Go and Gn
21	SNA	Angle formed between S, N, and A point representing the antero-posterior position of the maxilla in relation to the anterior cranial base
22	SNB	Angle formed between S, N, and B point representing the antero-posterior position of the mandible in relation to the anterior cranial base
23	ANB	Angle formed between A point, Nasion, and B point representing maxilla-mandibular relation.
24	Co-A	Maxillary length measured from the Co point to point A
25	Co-Gn	Mandibular length measured from the Co point to Gn point
26	N-ANS	Upper anterior facial height
27	ANS-Gn	Lower anterior facial height
28	PP inclination	Angle between PP and perpendicular to Se-N’ line dropped at N’
29	Nasal length	Length of nose from N’ to Pr
30	Nasal depth	Horizontal distance between Ac and Pr, drawn parallel to true horizontal line
31	Cm-Lb angle	Nasal tip angle formed by the tangent to Cm and Lb point
32	UNLA	Upper nasolabial angle formed between the tangent to the lower border of nose and line passing through Sn parallel to the true horizontal plane
33	NFL	Nasofacial angle (angle formed between line joining N’-Pr and line joining G’-Pg’)

The investigators who performed the measurements and the statistician were blinded to the group allocation and were provided with the coded sample. The codes are known to the principal investigator. Measurements were performed by two observers who were blinded to each other. To test the reliability of the measurements, 40 randomly selected cephalograms were retraced one week later by the same orthodontists who were blinded to their previous measurements, and the reliability of the parameters was examined using the intraclass correlation coefficient (ICC) for inter- and intra-observer reliability. The method error (ME) was assessed using Dahlberg’s test, which revealed ME values below 1 for linear dimensions (0.69 mm) and above 1^0^ for angular dimensions (0.75^0^). The ICC indicated strong reliability (.86 for inter-observer and .92 for intra-observer). There were no statistically significant variations in repeated measurements across all the variables, thereby refuting the existence of systematic errors.

Statistical analysis

The collected data were subjected to statistical analysis using IBM SPSS Statistics for Windows, Version 22 (Released 2013; IBM Corp., Armonk, New York, United States). The normal distribution of the data was examined using the Shapiro-Wilk test, and the data was found to be non-normally distributed. Therefore, non-parametric tests such as Mann-Whitney and Kruskal-Wallis tests were applied for the parameters. The compilation and presentation of sociodemographic variables and participant responses were achieved using frequency distributions and tables. Parametric data is presented as mean ± standard deviation (SD). The first objective of this study was to assess the correlation between different variables such as nasal morphology and skeletal parameters. This was assessed using Spearman’s correlation analysis for all parameters except the type of skeletal pattern for which the Eta correlation test was used. Multinomial logistic regression analysis was used to study various parameters as predictors of the type of skeletal pattern, where the class I skeletal pattern was considered as a reference. The second objective was to assess sex differences in nasal morphology, for which the Mann-Whitney U test for the parameters was used between the groups. The third objective was to assess the nasal morphology in different skeletal malocclusions, for which the Kruskal-Wallis test for the parameters was used. The fourth objective was to assess the optimal cut-off values for UNLA for different skeletal malocclusions, for which receiver operating characteristic (ROC) curves were used based on the Youden index and the area under the curve (AUC). Records with missing data were also excluded. The level of significance was set at P ≤0.05. Bonferroni corrections were applied for multiple variables using the Mann-Whitney test and Kruskal-Wallis test to reduce type 1 errors. Accordingly, a p-value ≤ 0.003 was considered statistically significant for comparisons using the above-mentioned tests.

## Results

Baseline data

The mean age of Group 1 patients was 21.02±1.59 years, comprising 30 (18.51%) males and 38 (23.46%) females, 20 (12.34%) patients had a low or horizontal growth pattern, 31 (19.14%) patients had an average growth pattern and 17 (10.49%) patients had a high or vertical growth pattern. Sixty-eight (41.97%) skeletal class II patients had a mean age of 20.90±1.69 years, comprising 32 (19.75%) males and 36 (22.22%) females, 18 (11.12%) patients had a horizontal growth pattern, 30 (18.51%) patients had an average growth pattern and 20 (12.34%) patients had a vertical growth pattern. Twenty-six (16.06%) skeletal class III patients had a mean age of 20.9±1.64 years, comprising 15 (9.27%) males and 11 (6.79%) females, six (3.71%) patients had a horizontal growth pattern, 12 (7.41%) patients had an average growth pattern and eight (4.94%) patients had a vertical growth pattern (Table 2).

**Table 2:** Basic characteristics of the study sample. SD: Standard deviation; Data presented in n(%)

Variables	Class I	Class II	Class III	Total
Participants n (%)	68 (41.97%)	68 (41.97%)	26 (16.06%)	162 (100.00%)
Age (years) Mean±SD	21.02±1.59	20.90±1.69	20.91±1.64	20.99±1.63
Gender (n%)				
Male	30 (18.51%)	32 (19.75%)	15 (9.27%)	77 (47.53%)
Female	38 (23.46%)	36 (22.22%)	11 (6.79%)	85 (52.47%)
Growth pattern (n%)				
Low	20 (12.34%)	18 (11.12%)	6 (3.71%)	44 (27.17%)
Average	31 (19.14%)	30 (18.51%)	12 (7.41%)	73 (45.06%)
High	17 (10.49%)	20 (12.34%)	8 (4.94%)	45 (27.77%)

Primary outcome

The UNLA showed a moderate positive correlation with the SNA angle and a moderate negative correlation with the SNB and PP inclination angles, which were statistically significant. This implies that the UNLA angle increases in the prognathic maxilla with a downward PP inclination and a retrognathic mandible. The NFL angle showed a strong positive correlation with the SNA angle and a strong negative correlation with the SNB and PP inclination angles, which was statistically significant. This implies that the NFL angle increased in the prognathic maxilla with a downward PP inclination and a retrognathic mandible. The Cm-Lb angle showed a very weak negative correlation with Co-A and Co-Gn, a strong negative correlation with SNA and ANB angles, and a strong positive correlation with SNB and PP inclination angles, which was statistically significant. This implies that the Cm-Lb angle increased in short maxillary and mandibular lengths, retrognathic maxilla with upward inclination of the PP, Class III skeletal pattern, and prognathic mandible. The nasal length and depth showed a very weak positive correlation with the lower anterior facial height, which was statistically significant. This implies that the increased nasal length and depth were observed in patients with an increased lower anterior facial height. The nasal parameters were not affected by changes in the upper anterior facial height or growth pattern of the individual. The findings are summarized in Table 3.

**Table 3:** Correlation between nasal parameters and skeletal parameters (Spearman correlation). *p<0.05: Significant; Very weak correlation: 0.0<∣r∣<0.20; Weak: 0.2≤∣r∣<0.4; Moderate: 0.4≤∣r∣<0.6; Strong: 0.6≤∣r∣<0.8; Very Strong: 0.8≤∣r∣≤1.

Skeletal parameters	Correlation	Nasal parameters				
		UNLA^o^	NFL^o^	Cm-Lb^o^	Nasal length (mm)	Nasal depth (mm)
Type of skeletal pattern	Eta	0.198	0.077	-0.080	0.044	-0.080
	p	0.011*	0.332	0.302	0.574	0.309
N-ANS (mm)	r	0.025	0.101	-0.114	-0.023	-0.022
	p	0.754	0.203	0.147	0.775	0.784
ANS-Gn (mm)	r	0.052	0.048	-0.123	0.273	0.274
	p	0.508	0.543	0.118	0.001*	0.001*
Co-A (mm)	r	0.057	0.094	-0.258	-0.073	-0.074
	p	0.474	0.237	0.001*	0.356	0.352
Co-Gn (mm)	r	0.038	0.091	-0.294	-0.007	-0.007
	p	0.631	0.248	0.001*	0.931	0.931
SNA^o ^	r	0.512	0.79	-0.654	-0.079	-0.078
	p	0.001*	0.001*	0.001*	0.319	0.321
SNB^o ^	r	-0.472	-0.772	0.677	0.04	0.04
	p	0.001*	0.001*	0.001*	0.613	0.612
PP inclination^o ^	r	-0.504	-0.77	0.636	0.058	0.058
	p	0.001*	0.001*	0.001*	0.465	0.466
ANB^o ^	r	0.052	0.782	-0.684	-0.023	-0.023
	p	0.509	0.001*	0.001*	0.772	0.772
SN-GoGn^o^	r	0.125	0.01	-0.01	0.088	0.088
	p	0.112	0.903	0.902	0.266	0.264

The results of the multinomial logistic regression analysis showed that none of the nasal and skeletal parameters was a significant predictor of the type of skeletal pattern (Table 4).

**Table 4:** Multinomial regression analysis for various parameters. p>0.05: Non-significant; PP: palatal plane

Variables	Class II related to Class I		Class III related to Class I	
	Coefficient	p-value	Coefficient	p-value
UNLA^o ^	0.485	0.994	0.242	0.996
Nasal depth (mm)	-44.677	0.995	17.020	0.998
Nasal length (mm)	29.866	0.995	-11.310	0.998
Cm-Lb^o^	-0.042	0.999	0.336	0.995
NFL^o^	1.158	0.985	-0.101	0.999
ANB^o^	1.491	0.995	-4.207	0.981
SNB^o^	-1.070	0.994	0.736	0.995
SNA^o^	1.836	0.989	-0.748	0.995
SN-GoGn^o^	0.118	0.996	0.092	0.997
Co-A (mm)	0.160	0.993	-0.033	0.999
Co-Gn (mm)	-0.366	0.984	-0.136	0.994
ANS-Gn (mm)	-0.218	0.994	-0.028	0.999
N-ANS (mm)	0.249	0.996	-0.385	0.996
PP inclination^o^	-2.063	0.987	0.242	0.996

Secondary outcome

The statistically significant gender differences were observed in nasal length, nasal depth and ANS-Gn, where males had a significantly larger nasal length (41.31±3.60 mm), nasal depth (27.54±2.39 mm), and lower anterior facial height (54.74±4.71 mm) than females, as shown in Table 5.

**Table 5:** Gender-wise comparison of nasal and skeletal parameters in the study sample by Mann-Whitney U test. *p-value ≤ 0.003: Significant; Data represented in the form of mean±SD.

Variables	Gender	Mean±SD		Confidence interval at 95%		p-value
				Upper limit	Lower limit	
Nasal parameters						
UNLA^o^	M	20.42±3.23		21.16	19.69	0.904
	F	20.71±4.26		21.63	19.79	
NFL^o^	M	38.72±4.99		39.86	37.59	0.645
	F	39.12±4.85		40.17	38.08	
Cm-Lb^o^	M	35.01±4.27		35.98	34.04	0.15
	F	33.96±3.68		34.75	33.17	
Nasal length (mm)	M	41.31±3.60		42.12	40.49	0.001*
	F	37.14±3.22		37.83	36.44	
Nasal depth (mm)	M	27.54±2.39		28.08	26.99	0.001*
	F	24.75±2.14		25.21	24.29	
Skeletal parameters						
ANB^o^	M		3.29±3.18	4.02	2.57	0.656
	F		3.72±3.00	4.37	3.08	
SN-GoGn^o^	M		32.50±7.64	34.24	30.77	0.661
	F		32.02±6.39	33.40	30.64	
N-ANS (mm)	M		42.81±2.64	43.41	42.21	0.043
	F		43.67±2.57	44.22	43.11	
ANS-Gn (mm)	M		54.74±4.71	55.81	53.67	0.001*
	F		51.08±3.60	51.86	50.30	
Co-A (mm)	M		82.58±6.74	84.11	81.05	0.159
	F		85.21±8.17	86.97	83.44	
Co-Gn (mm)	M		103.46±14.7	106.79	100.11	0.112
	F		107.03±15.4	110.71	104.06	
SNA^o^	M		82.37±3.53	83.18	81.57	0.369
	F		82.85±3.25	83.56	82.15	
SNB^o^	M		79.00±3.16	79.71	78.28	0.625
	F		78.68±2.79	79.28	78.07	
PP inclination^o^	M		86.90±3.04	87.59	86.21	0.788
	F		86.87±3.12	87.54	86.19	

The different skeletal malocclusions showed statistically significant differences in angular parameters, such as UNLA, NFL, Cm-Lb angle, SNA, SNB, ANB, and PP inclination angle, and linear parameters, such as N-ANS and Co-Gn. Increased UNLA, NFL, ANB, and SNA angles have been observed in the skeletal class II pattern but decreased in the skeletal class III pattern. This showed that skeletal class II patients had been associated with an upward-tipped large nose, prognathic maxilla, small retrognathic mandible, and downward-tipped PP, whereas skeletal class III was associated with a downward-tipped small nose, retrognathic maxilla, large prognathic mandible, and upward-tipped PP (Table 6).

**Table 6:** Comparison of nasal and skeletal parameters in different malocclusions by the Kruskal-Wallis test. *p-value ≤ 0.003: Significant; Data represented in form of mean±SD.

Variables	Skeletal pattern	Mean± SD	Confidence interval at 95%		p-value
			Upper limit	Lower limit	
Nasal parameters					
UNLA^o^	Class 1	19.29±3.46	20.13	18.45	0.001*
	Class 2	22.94±2.93	23.65	22.23	
	Class 3	16.76±3.16	19.04	16.49	
NFL^o^	Class 1	37.01±2.14	37.53	36.49	0.001*
	Class 2	43.44±2.82	44.12	42.75	
	Class 3	32.19±3.06	33.42	30.95	
Cm-Lb^o^	Class 1	34.88±2.66	35.52	34.23	0.001*
	Class 2	31.85±2.50	32.45	31.24	
	Class 3	40.19±3.74	41.70	38.68	
Nasal length (mm)	Class 1	39.55±3.86	40.49	38.62	0.488
	Class 2	38.75±4.11	39.74	37.75	
	Class 3	38.96±4.01	40.58	37.34	
Nasal depth (mm)	Class 1	26.36±2.57	26.99	25.74	0.493
	Class 2	25.83±2.74	26.49	25.16	
	Class 3	25.97±2.67	27.05	24.89	
Skeletal parameters					
ANB^o^	Class 1	3.20±0.61	3.35	3.05	0.001*
	Class 2	6.17±0.91	6.39	5.95	
	Class 3	-2.57±0.50	-2.37	-2.78	
SN-GoGn^o^	Class 1	32.05±7.54	33.88	30.23	0.655
	Class 2	32.27±6.94	33.96	30.59	
	Class 3	32.69±5.76	35.02	30.36	
N-ANS (mm)	Class 1	44.13±2.89	44.83	43.43	0.001*
	Class 2	43.07±2.33	43.64	42.50	
	Class 3	41.50±1.47	42.09	40.90	
ANS-Gn (mm)	Class 1	52.61±4.52	53.71	51.52	0.225
	Class 2	53.50±4.78	54.65	52.34	
	Class 3	51.57±3.72	53.08	50.07	
Co-A (mm)	Class 1	84.25±6.87	85.91	82.58	0.032
	Class 2	85.10±8.71	87.21	82.99	
	Class 3	80.23±4.99	82.25	78.21	
Co-Gn (mm)	Class 1	112.77±14.88	116.38	109.11	0.001*
	Class 2	94.69±6.99	96.38	92.99	
	Class 3	114.84±14.35	120.64	109.04	
SNA^o^	Class 1	81.14±1.42	81.49	80.80	0.001*
	Class 2	86.02±0.91	86.25	85.80	
	Class 3	77.61±1.65	78.28	76.94	
SNB^o^	Class 1	79.45±1.52	79.82	79.0	0.001*
	Class 2	76.22±0.68	76.38	76.05	
	Class 3	84.03±1.24	84.54	83.53	
PP inclination^o^	Class 1	87.94±1.58	88.32	87.55	0.001*
	Class 2	83.97±0.84	84.17	83.76	
	Class 3	91.76±1.14	92.23	91.30	

**Figure 4:**
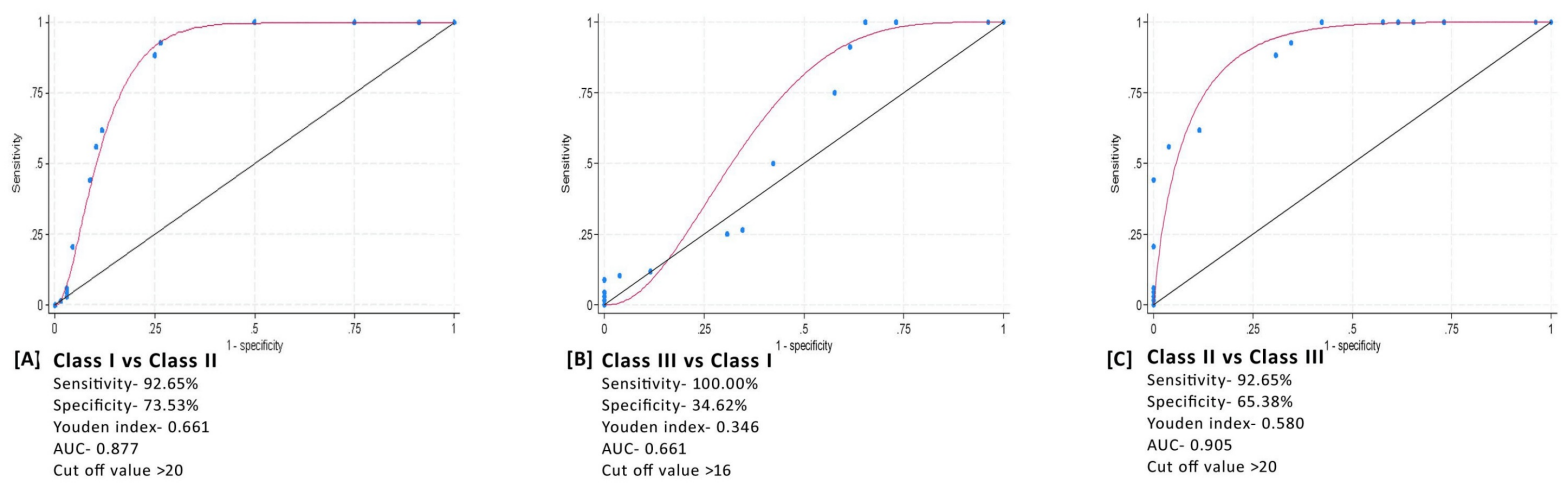
Receiver operating characteristic (ROC) curves for the upper nasolabial angle in different skeletal malocclusions.

ROC analysis revealed that UNLA is a good variable to differentiate between skeletal class I and II malocclusion, with an optimal cut-off value of ≥20^0^ at a Youden index of 0.662, AUC of 0.877, specificity of 74%, and sensitivity of 93%. It is also a good variable to differentiate between skeletal class II and III malocclusion, with an optimal cut-off value of ≥20^0^ at a Youden index of 0.58, an AUC of 0.905, a specificity of 65%, and a sensitivity of 93%. The optimal cut-off values indicate that if the UNLA value is ≤16^0^, there is a probability of a class III skeletal pattern; if the UNLA value is ≥ 20^0^, then there is a probability of a class II skeletal pattern; and if the UNLA value is between 16^0^ and 20^0^, then there is a probability of a class I skeletal pattern (Figure 4).

## Discussion

Improvement in facial aesthetics is the main goal of orthodontic treatment, where the nose is located in the center of the face, and forms a very important part of diagnosis and formulating a treatment plan that can be camouflage treatment or orthognathic surgery. Rhinoplasty is routinely performed as a cosmetic procedure to improve facial aesthetics; therefore, it is essential to assess the factors that can influence nasal morphology, particularly the UNLA, which has been associated with upward or downward tipping of the nose. However, this component has not yet been studied in detail. According to a study by Naini et al., if the UNLA is more than 33^0^ or less than 8^0^, it is perceived as unattractive and requires a surgical procedure (rhinoplasty) for correction; therefore, it forms an essential component of facial aesthetics [9]. The present study divided the study sample into 1:1:0.4 (class I: class II: class III). This was due to the lower prevalence of class III skeletal relationship in the Indian population which is in accordance with previous studies [10,11].

The results of the present study revealed that the UNLA and NFL angles are affected by the sagittal position of the maxilla and mandible, and the inclination of the PP. It is increased in patients with prognathic maxilla and retrognathic mandible (skeletal class II), and individuals with downward inclination of the PP. This finding indicated that the upward nasal tip and larger nose were associated with an underlying class II skeletal pattern in the individual. The upper anterior facial height was lower in skeletal class III patients with upward tipping of the PP. They also had a retrognathic maxilla and prognathic large-sized mandible compared to the increased upper anterior facial height with downward inclination of the PP in skeletal class II patients [10].

Latham proposed that the nasal septum serves as an initiating mechanism, facilitating the anterior movement of the premaxilla and maxilla through the septo-premaxillary ligaments, explaining the prevalence of a large nose in class II patients with maxillary prognathism found in the present study [12]. Kim et al. found that inadequate development of the nasal septum, combined with reduced anteroposterior growth of the maxilla, results in an upward shift of the front part of the maxilla, demonstrating a significant association between nasal growth and tilt of the maxillary plane [13]. These findings are in agreement with those of a study conducted by Faryal et al. [14]. In contrast, Prasad et al. observed that the upward tipping of the nose was associated with the upward inclination of the palatal plane [15]. The reason for this finding might be the different measurement landmarks used to measure the UNLA. The UNLA was measured as the angle between the lower border of the nose and the Frankfort-horizontal (FH) plane, whereas in our study, it was taken as the angle between the lower border of the nose and the true horizontal plane, which has been reported as a more reliable plane than the FH plane [16]. However, none of the nasal parameters were reliable predictors of the underlying skeletal pattern of the individual.

In our study, it was observed that the optimal cut-off values for the UNLA angle in class II patients were ≥ 20^0^, 16-20^0^ for skeletal class I, and ≤ 16^0^ for skeletal class III patients. The average value of UNLA in class I was found to be 19.29±3.46^0^, 22.94±2.93^0^ in class II, and 16.76±3.16^0^ in class III. According to Naini et al., the ideal value of the UNLA is 12-24^0^, however, they did not evaluate this angle for different skeletal patterns [9]. Significant sex differences were noted in the UNLA parameters. The UNLA was not affected by the length of the maxilla and mandible or by vertical parameters. This is in accordance with the results of a previous study [7].

The nasal length and depth showed a weak positive correlation with lower anterior facial height in the present study, indicating that individuals with a large lower facial height had a long and prominent nose. This finding is in agreement with those of Nehra and Sharma [3]. Significant sex differences were observed in nasal length and depth in our study, with males having a longer and more prominent nose than females. This was in accordance with a previous study [17]. The results of the present study revealed that skeletal class III patients had an upward canted nasal tip (increased nasal tip angle) compared to class II and class I patients, which was in accordance with the results of previous studies by Faryal et al. [14] and Prasad et al. [15]. Our study did not report any correlation between nasal parameters and the growth pattern of the individual.

Strengths and clinical implications

In a systematic review conducted by Sahoo et al. [18], previous studies examined the relationship between nasal and skeletal parameters without categorizing samples based on different skeletal patterns [3,15]. Unlike these studies, the current study classified the study sample into distinct skeletal patterns and analyzed various nasal and skeletal parameters as potential indicators of skeletal malocclusion. To date, no study has determined the optimal cut-off values for UNLA using ROC curves in various skeletal malocclusions. Modifying teeth and jaws alone may have a detrimental effect on the harmonious aesthetic balance of the nasal facial/dental complex. For example, alterations in the lips resulting from orthodontic interventions aimed at correcting protruding incisors can lead to an increased visibility of the nose. In cases involving the prominent nose, heightened UNLA, and nasal tip angle, caution should be exercised when deciding on orthodontic retraction of the teeth, with the aim of minimizing retraction without compromising facial aesthetics. Choosing a non-extraction approach could be advantageous in such scenarios. Surgical interventions on the maxilla and mandible that impact the soft tissues, chin, and lower lip can also affect the prominence of the nose. Hence, it is crucial to include rhinoplasty in the treatment plans for orthognathic cases. The significance of the nose in facial identification and its involvement in various forensic investigations underscores the importance of orthodontists' contributions to nasal morphology assessment, which can significantly aid in the accurate reconstruction of facial characteristics in forensic facial approximation. The nose undergoes subtle changes over time, particularly from childhood to adulthood. While many facial features change with age, the general shape and proportions of the nose remain relatively consistent. Forensic experts can analyze nasal morphology in photographs taken at different stages of life to establish identity. The nose does not experience drastic changes, making it a reliable feature for comparison. In cases of mass disasters or severe decomposition where facial features are difficult to recognize, the nose's skeletal structure (particularly the nasal bones and surrounding maxillary bones) can be crucial for reconstructing the individual's appearance.

Limitations

The retrospective nature of this study affects the generalizability of the results. The study followed an unequal sample distribution in three skeletal classes due to the lower prevalence of class III skeletal patterns in the Indian population. The present study used lateral cephalograms for analysis; however, 3-D CBCT could provide more precise information on high-resolution 3-D images [19]. Moreover, this was a single-center study; therefore, future prospective multicenter studies with larger sample sizes should be conducted to validate the results of the present study.

## Conclusions

Within the limitations of the present study, it was concluded that the UNLA was significantly correlated with the sagittal position of the maxilla and mandible, and PP inclination. It was found to increase in the skeletal class II pattern with a downward inclination of PP. Patients with skeletal class III malocclusion had an upward-canted nasal tip with a decreased upper nasolabial angle. In our study, it was observed that the optimal cut-off values for the UNLA angle in class II patients were ≥ 20^0^, 16-20^0^ for skeletal class I, and ≤ 16^0^ for skeletal class III patients. None of the skeletal and nasal parameters were reliable predictors of the skeletal pattern type. The statistically significant gender differences were observed in the nasal length, nasal depth, and lower anterior facial height, where males had a significantly larger nasal length (41.31±3.60 mm), nasal depth (27.54±2.39 mm), and lower anterior facial height (54.74±4.71 mm) than females.
